# Implementing an Action Over Inertia Group Program in Community Residential Rehabilitation Services: Group Participant and Facilitator Perspectives

**DOI:** 10.3389/fpsyt.2021.624803

**Published:** 2021-02-02

**Authors:** Erin F. Rees, Priscilla Ennals, Ellie Fossey

**Affiliations:** ^1^The Royal Melbourne Hospital – North Western Mental Health, Melbourne, VIC, Australia; ^2^Neami National, Preston, VIC, Australia; ^3^Department of Occupational Therapy, Social Work and Social Policy, La Trobe University, Melbourne, VIC, Australia; ^4^Department of Occupational Therapy, Monash University, Melbourne, VIC, Australia; ^5^Living With Disability Research Centre, La Trobe University, Melbourne, VIC, Australia

**Keywords:** Action Over Inertia, psychosocial rehabilitation, recovery oriented practice, activity participation patterns, group interventions, community care unit, qualitative research

## Abstract

**Introduction:** A time-use focused intervention, Action Over Inertia (AOI) designed to address restricted activity patterns and support recovery, was adapted for use in Australian community residential mental health services.

**Method:** Qualitative case study research explored the use of AOI groups across three Community Care Units from the perspectives of group participants with enduring mental illness and group facilitators. Fifteen interviews were conducted: five group participants were interviewed twice 4 weeks apart, and five group facilitators on completion of the group intervention. Interview data were analyzed thematically using constant comparative methods.

**Findings:** Two overarching themes, “Making Change” and “Facilitating Change” were identified. Efforts to make change in their lives were supported by participants recognising the value of personally meaningful activities for well-being and of activity experiences that fostered hope and recovery, whereas a sense of “stuckness,” time for activities and life events could disrupt “getting me going.” For the facilitators, facilitating change involved recognizing inertia as a challenge; getting people going; and looking at how AOI intervention works to impact inertia.

**Conclusion:** AOI in a group format supports participants to identify barriers to more active living; to appreciate how time-use and well-being interrelate; and to reframe and take steps to overcome inertia. Further research should evaluate AOI groups as a means of providing individualized support for activity re-engagement as part of recovery oriented mental health rehabilitation.

## Introduction

Recovery informed policies have become influential in mental health services internationally (1). For services developed within the context of de-institionalization to provide community residential care and rehabilitation for people with enduring mental illness, recovery oriented frameworks have necessitated a shift from expert-driven rehabilitative approaches to recovery informed practices that value and respect individuals' views and lived experiences, rights and choices (1–3). Key differences include that recovery informed practices are person driven, rights-based and foster choice and self-determination, self-chosen goals and directions; they foster hope, collaborative partnerships, and engagement of support networks; and focus on strengths, developing capabilities and community participation (4). An evidence base informed by experiential and scientific research for psychosocial interventions using approaches focused on individualized support, skill development, and peer involvement has developed to support people in their personal recovery (4, 5). Most notably in the vocational domain, a strong evidence base for individualized support to enable engagement in education and employment has developed (6), while healthy lifestyle interventions that typically target physical activity are also increasingly recognized as important in recovery (7, 8). Furthermore, Strengths and recovery informed approaches also emphasize the provision of supports to identify and pursue self-chosen goals in life domains broadly (4, 9, 10).

Involvement in self-chosen, personally and culturally meaningful activities is widely identified as a factor contributing to recovery and well-being (11, 12). Yet, adults with enduring or severe mental illness (SMI) are disadvantaged in housing, employment, social and financial status, as well as having poorer health than the rest of the population, so that overcoming social exclusion and reclaiming a meaningful life is a significant challenge (1). How people spend their time is a recognized indicator of health and well-being, given various features of activities engaged in a temporal context can promote or compromise health (13). Hence, time use data can be used to describe the distribution and correlates of health-enhancing activity patterns relevant to public health; it can also provide important information in mental health care contexts about how people with enduring mental illness spend their time during the day (13, 14). Time-use studies involving adults with first-episode psychosis and enduring or severe mental illness (SMI) indicate their activity patterns are frequently characterized by few active, meaningful or socially valued forms of occupation (15–17). Some work to develop time-use informed measures of activity participation in mental health care has occurred (18, 19). However, time-use informed interventions that specifically address broad disruptions in activity patterns and development or re-engagement in meaningful and satisfying activity patterns are less well-developed in comparison to other psychosocial interventions (20–22).

Time use, activity engagement, patterns of participation and their links to health and well-being are longstanding concerns of occupational therapy (14, 23). While there is evidence showing the need for interventions addressing activity patterns and engagement in an individualized, collaborative manner to promotes recovery (24), further establishment of their outcomes in terms of changes in occupational engagement and activity patterns, as well as recovery and well-being, is required (21). An important step forward has been the development of manualized interventions that address disruptions in activity patterns and occupational engagement (21), such as Action Over Inertia (AOI) (25) and Balancing Everyday Life (BEL) (20). Both are informed by a time-use perspective and situated in relation to contemporary recovery oriented frameworks for mental health practice.

Developed by occupational therapists in Canada, Action Over Inertia (AOI) is a flexible workbook-based, time-use intervention for use in collaboration with people experiencing challenges of everyday living with severe mental illness (25, 26). This approach acknowledges that living with mental illness is challenging, but also takes the view that these struggles need not prevent an individual from engaging in personally meaningful activities associated with recovery, health and well-being benefits (25). These benefits of activity engagement are not limited to but may include expressing one's own goals and values; developing one's skills or knowledge; improving mental and physical well-being; interacting with others; contributing in one's community; experiencing pleasure and satisfaction (25, 27). In this sense, AOI aligns with recovery oriented practice through providing tools and resources for supporting individuals to build activity patterns that enable fulfilling lives irrespective of the presence of ongoing mental ill-health. Based on a small-scale prospective randomized controlled pilot study to investigate the use of AOI with individuals with SMI receiving assertive community treatment, Edgelow and Krupa (28) reported that individuals receiving the AOI intervention made positive changes in how much time was spent in activities other than sleep. Participants also commented positively on their experience of AOI and changes made in their daily lives. While their study indicated that AOI is relevant and useful, Edgelow and Krupa recommended further research investigate its implementation in different settings and formats. In comparison, BEL is an evidence-informed 12 session group-based and lifestyle redesign intervention for people using community mental health services developed in Sweden. It focuses on accomplishing a satisfying amount and variation in activities, meaning, healthy living, work-related, leisure, relaxation and social activities, and supporting recovery (20). Improved activity engagement and activity levels were demonstrated in a recent randomized controlled trial comparing BEL with usual care (20). Group leaders' and participants' perspectives of the BEL intervention have also been reported, suggesting the value of joining with others and group support to bring about meaningful activity changes in daily life (29, 30).

In Australian community mental health settings, occupational therapists have begun using AOI in a group format to not only promote understanding of the contribution of activity participation in recovery and well-being, but also to foster group support for self-development and effecting change. Little is known about the delivery or experience of AOI in group formats. Yet, an important dimension of therapeutic effectiveness is how interventions are experienced, the degree to which they are impactful and the change processes involved from the perspectives of those accessing services (24, 31). This paper reports qualitative case study research of a group-based Action Over Inertia intervention involving adults with enduring mental illness in Australian community residential rehabilitation programs, the overall aim of which was to understand its use in this setting from the viewpoints of group participants and facilitators.

## Materials and Methods

### Design

This qualitative naturalistic case study was informed by an interpretive standpoint (32) focused on understanding the individual and shared meanings of engagement in Action Over Inertia (AOI) groups. It addressed two main aims: the first was to investigate the experience of Action Over Inertia (AOI), its impacts on identifying barriers to occupational engagement, improving participation in meaningful activities, and achieving a sense of recovery from the perspective of adults with SMI. The second aim was to describe AOI group facilitators' views and experience of facilitating the intervention. Qualitative case study research methodology was chosen since it is well-suited to describing and understanding specific programs or interventions within their “real world” or naturalistic settings; and is useful for exploring processes involved in program implementation that can be difficult to study experimentally (32–34). Qualitative case study research should also have pre-defined boundaries that may address time, place and individuals, defining what is and is not studied (32, 35). Hence, a qualitative naturalistic case study approach lends itself to investigating a new practice or service development, such as the implementation of Action Over Inertia, from the perspective of people accessing and delivering it within a particular health care context.

### Setting

**Figure 1:**
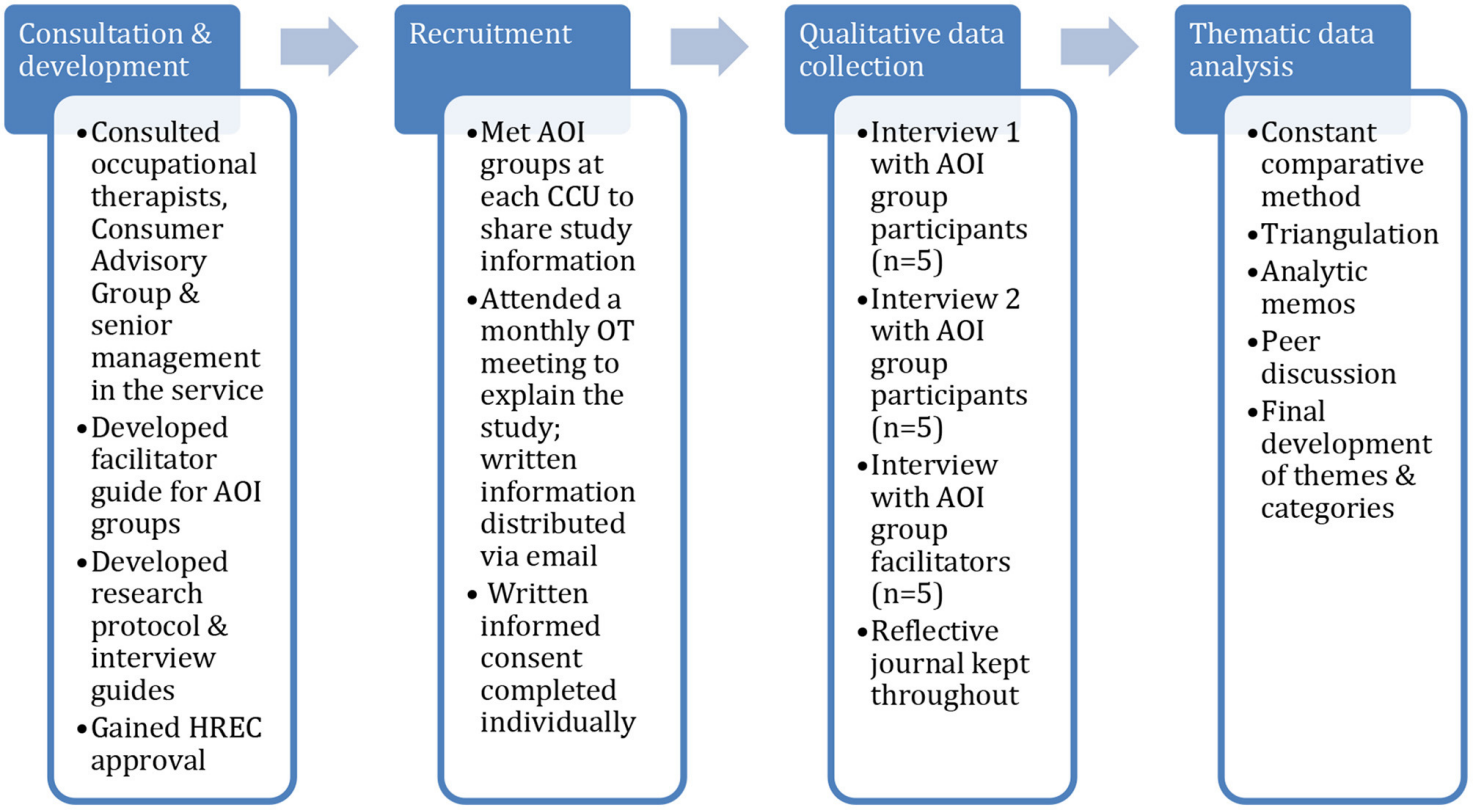
Research process.

This qualitative case study was bounded to three clinically operated community residential services, known as Community Care Units (CCU), within one health authority in metropolitan Melbourne, Australia, where training in Action Over Inertia had recently been provided for occupational therapists of the mental health services. As part of the public mental health service system, the CCU services were originally established to provide accommodation, rehabilitation and clinical care for people moving out of hospitals in the context of de-institionalization, many of whom have since moved to other community housing (3). Generally, CCUs provide 24-h clinical care and rehabilitation support using a multidisciplinary approach, with an emphasis on transition to community living and on facilitating recovery (2, 3). The three CCUs in this study were located in residential neighborhoods and accommodated up to 20 adults experiencing prolonged mental illness in clustered 2-3 bed self-contained units with some communal facilities, and an on-site multidisciplinary mental health staff team, as is typical of CCUs (3). For international comparison, these residential services may be best classified as Type 1 using the STAX-SA taxonomy developed in England (i.e., congregate accommodation, on-site clinical staff, high support) (36), although the focus on moving to other housing options, albeit not within a set time period, overlaps Type 2 in this taxonomy (3). The three CCUs each offered a five-day a week rehabilitation group program facilitated by occupational therapists, which included a variety of groups focused on psychoeducation, symptom management, activities of daily living, exercise and physical health. Given these CCU group programs lacked a focus on time use and well-being, and on identifying barriers to more active living and engagement, they were purposively selected to trial using AOI in a group format as part of the occupational therapists' ongoing work with residents. Local adaptation of the AOI workbook (25) for use in a group format, development of the research protocols and the interview questions each involved extensive consultation with these occupational therapists, the Consumer Advisory Group, and senior management of the mental health services. An overview of the research process is illustrated in Figure 1.

### Group-Based Action Over Inertia

The AOI intervention was adapted for use in a group format over up to eight sessions by the first author, an occupational therapist with training in using AOI, in consultation with occupational therapists and the Consumer Advisory Group. Both the AOI manual (25) and a locally developed AOI facilitator guide were used by occupational therapists in planning the group sessions run at the three CCUs. The groups were run with two facilitators per group. Table 1 describes the group session aims and AOI worksheets and resources used to support exploration of each session topic. The group program was individualized, so that the groups explored the topics at varying pace or with varied emphasis, depending on issues most relevant to group members. The three group programs also differed in length, being run over five to eight sessions, with all group participants involved in at least four sessions.

**Table 1:** Overview of AOI group sessions, AOI worksheets, and resources used.

**Group session**	**Aim and focus**	**AOI worksheets**	**AOI resources**
I	Introduce AOI and reflect on current activity patterns. Develop awareness of time-use patterns.	1.3 My current activity patterns 1.4 Benefits of my current activities 2.1 Daily time use log	
II	Collecting information about current activity patterns Reflecting on current activity patterns	2.1 Daily time use log 2.2 My daily time use 2.3 Considering the balance of my activities^*^ 2.4 Am I getting enough physical activity?^*^ 2.5 My daily routine and structure^*^	2.2 Daily activity (examples of diverse activities)
	Increase awareness of different meanings and types of activities. Creating individual definitions of occupation.	2.6 Finding meaning in my activities^*^ 2.7 Satisfaction with activities^*^	2.2 Daily activity
III	Continued from session two with greater focus on reflection of past, present and future time use.	2.2 My daily time use 2.6 Finding meaning in my activities 2.7 Satisfaction with activities^*^ 2.8 Social interaction through activities^*^ 2.9 Accessing my community^*^	2.1 Levels of activity engagement
	Identify different states of occupational balance/imbalance. Consider “just right” balance in preparation for areas of change.	2.10 Activity Engagement Measure^*^	
IV	Introduce quick activity changes and think of small changes to increase general activity engagement. Plan for short-term action.	3.1 Record of activity experiments	3.1 Some ideas about quick activity changes
	Identify different states of occupational balance/imbalance. Consider “just right” balance in preparation for areas of change.	As above	As above
V	Reflect on recent engagement in activity experiments/quick activity changes, benefits and barriers. Identify problem solving strategies to overcome barriers and education to support participation.	2.6 Finding meaning in my activities 3.1 Record of activity experiments 4.1 Health and wellbeing benefits of my current activities^*^	4.2 One activity, many benefits 4.3 Making clear the benefits of activities 4.4 Recovery benefits of activity participation
VI	Continue session five as above		
	Develop clear plans for change. Identify potential challenges preventing activity engagement. Focus on health and wellbeing benefits associated with activity participation.	2.10 Activity Engagement Measure^*^ 5.1 Preparing for changes in activity participation^*^ 5.3 Planning for activity change^*^	5.2, 5.3
VII	Continue session six as above		
	Develop clear plans for change. Reflect on previous worksheets.	3.1 Record of activity experiments 5.1 Preparing for changes in activity participation 5.2 Prioritizing plans for activity change^*^ 5.3 Planning for activity change 5.4 Giving shape to plans for activity change^*^	3.1 Some ideas about quick activity changes
VIII	Reflect on what has been learnt in the sessions. Focus on current activity patterns (interest and meaning), balance, barriers to change and ways to address them.	5.3 Planning for activity change	5.1 Managing challenges to activity change

* *Asterisk indicates an AOI worksheet introduced in one CCU group, but not all three at this same time point in the AOI group program*.

### Sampling and Recruitment

Qualitative sampling aimed to purposively seek perspectives of Action Over Inertia (AOI) group participants and group facilitators on the basis that the views and experiences of both are important to the above study aims (37). Information about the study was distributed to all residents participating in AOI groups at the three CCUs and all AOI group facilitators, and it was made clear that participation in the research was not a condition of their involvement in AOI groups. The study was explained and written informed consent completed with each person who agreed to participate. The AOI group participants were reimbursed AUD $20 per interview for their time and expertise.

### Participants

Ten participants were recruited, including five AOI group participants and five group facilitators. The AOI group participants were aged 32–55 (mean 42 years); and had been residing at the CCU for 3–18 months (mean 9.4 months); four participants had a diagnosis of schizophrenia and the fifth person was diagnosed with schizoaffective disorder. This profile is consistent with that reported of CCUs elsewhere (3). The group facilitators included: four occupational therapists, two of whom were employed as senior clinicians and two in entry level positions with 1.5–15 years' practice experience (mean 6.6 years); and one final year occupational therapy student.

### Qualitative Data Collection

Qualitative data were gathered through 15 individual, in-depth, semi-structured interviews with the five AOI group participants and five group facilitators, each conducted by the first author. Interview questions were informed by knowledge of the AOI intervention, underpinning literature and consultations with the Consumer Advisory Group and senior occupational therapists in the mental health service. Two semi-structured interviews were completed with each AOI group participant. The first interviews were conducted at the beginning of the intervention and focused on participants' current activity patterns, views about recovery, and goals for participating in AOI group. The second interview occurred 4–6 weeks later and focused on participants' experiences of participation in an AOI group and reflections on the impact that it had on their daily routines and engagement in occupations. On completion of each AOI group program, the facilitators were each interviewed once. A semi-structured approach was used to explore AOI facilitators' experience of facilitating AOI in a group, any adaptations they would recommend to increase its usefulness, and their views of its impact on the group participants. The first author also used her knowledge of AOI to ask follow-up questions and encourage participants to expand on their experiences and ideas. Interviews were all conducted in a place and time of the participant's choice, and either audio-recorded with their consent or hand-written notes were made.

### Qualitative Analysis

The qualitative data analysis was guided by Braun and Clarke's (38) approach to thematic analysis. Interviews were transcribed professionally and by the first author to both ensure accuracy, and gain in-depth familiarity with the data. First, analysis of individual interviews by the first author included listening to each recording, thorough reading of each transcript, developing initial codes, and then identifying similarities and differences between codes using a constant comparative method, so as to group the data into meaningful categories that illuminated the subjective experiences of AOI group participants and facilitators, respectively (38, 39). Second, the categories were compared to draw out common characteristics across the interviews from AOI participants and facilitators, which helped identify emerging patterns, meanings and themes within the data (38). The second author independently reviewed and coded some transcripts; emerging categories and themes were then developed through discussion between the three authors.

Several trustworthiness strategies were used to enhance the quality of this study. These included consultations with occupational therapists and the Consumer Advisory Group in the mental health service during adaptation of AOI for use in a group format and development of the interview questions. In addition, reflective journaling by the first author was used during data collection and analysis processes to trace assumptions, decision-making processes and experiences, as well as peer supervision with the other authors, both of whom are experienced occupational therapy researchers. Data triangulation through inclusion of the viewpoints of group participants and facilitators about the same phenomenon about AOI groups at three sites strengthened the credibility of the themes (32, 39).

### Ethics Approval

The institutional Human Research Ethics Committees (HRECs) of Melbourne Health (2015.103) and La Trobe University Faculty of Health Sciences (LEG/13218) approved this research.

## Findings

Two overaching themes, “Making Change” and “Facilitating Change” were identified, representing perspectives of Community Care Unit (CCU) residents who participated in AOI groups and the group facilitators, respectively. Each is presented below, with direct quotes to illuminate the themes, and the use of pseudonyms as agreed with participants to ensure their confidentiality.

### Making Change

For AOI group participants, making change involved finding it hard to get themselves going, and recognizing the importance of doing so. As summarized in Table 2, they identified “stuckness,” time issues and other factors that restricted or disrupted their efforts to make changes and do what mattered to them. They also spoke of ways in which they were “getting themselves going,” and aspects of AOI that were supportive in doing so.

**Table 2:** “Making change”— a summary of key themes and descriptions.

**Key theme**	**Description**
It's hard to get myself going and things get in the way	Factors that prevented or got in the way of getting going: 1. Feeling of “stuckness.” 2. Finding time. 3. Barriers.
Getting myself going	Of central importance to getting myself going: 1. Recognizing the value of meaningful activities. 2. Doing things brings a sense of hope and recovery. Factors that enabled doing things that matter: 1. Understanding the relationship between activity, health and wellbeing. 2. Planning our time use. 3. Developing strategies to push through resistance and discomfort. 4. Quick activity exercises. 5. Experiencing the benefits of doing a little more. 6. Doing things with others. 7. Learning from others through talking.

#### It's Hard to Get Myself Going and Things Get in the Way

AOI group participants spoke of various factors that prevented or got in the way of them getting going, and doing what mattered to them. These factors varied between them and over time, and included: being out of the habit of routine activity or engaging in mainly passive activities; difficulties finding time amidst competing demands; life events that disrupted participation; the impacts of medications; and the paralyzing nature of feeling stuck.

AOI participants used terms such as “laziness,” “lack of motivation” and “inertia” to describe their “stuckness” and difficulty in getting going. For instance, James and John noted a “lot of bad habits,” doing relatively little, and fluctuating health issues as preventing them from getting going. In John's words:

Before CCU [I] was sitting on the couch, physical health and mental health was up and down which was stopping [me] from doing some activities. Didn't have motivation and some resistance to participation… I couldn't be bothered.

Further, James described the “stuckness” that he experienced as resistant to change, sharing that “nothing in the group helped with overcoming lack of motivation,” despite his thinking about what he might do to change:

Think all about how I could plan and change my days and my health and everything … think a lot more about my daily routine and the way that I very well could plan it a lot better.

In addition, while important in their recovery, some AOI participants indicated that time spent participating in the CCU program impacted their time to engage in other activities that were meaningful or mattered from their viewpoints. Thus, James spoke of his emerging understanding of his time use, competing demands on his time and how to manage it that he gained in the AOI group:

I wish I had more time. The CCU program takes up most of every single day … it's taking up nearly all of my time… Time is my issue here … I don't have enough time to do any of the stuff that I have to do … Pretty much after the AOI group, with those questions, [I discovered] my problem is time during the day, and all activities and chores I need to do in the day. I probably do think about my day more often, and need to plan it more better.

Craig similarly identified time use as a challenge, particularly as his days became more filled with activity:

It's more like finding time to do stuff. Like you work a bit and you've got heaps of work, and you have to sort of find spare time.

Several AOI participants reported that medication both restricted and enabled their participation in activities that mattered to them. For instance, medication could be a “barrier” to getting going and doing the things they wanted to do due to its sedating effect. In James' words, “[what] I am finding with the medication is I am having too much rest, not enough productivity.” He also noted his medication meant needing to be in bed early and so influenced his daily routines and what he could get done. In comparison, medication could also enable getting going and being engaged in activities, as John noted:

On no medication, I used to start hearing voices in me head … yeah, so, if I would not take the medication, I would not be here today.

The AOI group participants also spoke of other factors, including the ups and downs of life, that got in the way of their intentions to be more active. They described needing to accept that some of these factors were outside their sphere of influence, but also to acknowledge their impact on participation in daily activities. For example, John spoke of a friend's recent death making things “a bit rough,” as well as how a bike accident 3 years ago left him with a painful knee, meaning he “can't walk as much as I would like” and is more restricted in the activities that he would like to do. James too identified poor physical health as a barrier to his participation in meaningful activities.

#### Getting Myself Going

Despite the many challenges in getting themselves going, AOI participants described noticing many ways in which the AOI group impacted on their lives, helped get them going, and enabled their engagement in what mattered to them. Of central importance to getting themselves going, AOI participants identified: recognizing the value of meaningful activities; and how doing things brings a sense of hope and recovery. In particular, AOI group participants spoke of developing their understanding of connections between activity, health and well-being; planning their time use; developing strategies to push through resistance and discomfort; experiencing the benefits of doing a little more; doing things with others; and learning from others through talking.

Learning about activities and health, and about time use was a focus in the AOI groups that enabled participants to make connections they had not previously understood, or perhaps had forgotten. For example, Frank described previously being unable to recognize the importance and impact of meaningful activities and the resistance he experienced before participating in AOI, summing up his developing awareness of these factors within the group:

The inertia part we touched [on] today, and we were talking about the goals and how resistance was used by, within ourselves.

Further, for James, participation in AOI increased his awareness of what he could be doing, and helped him to make changes:

I definitely now wake up, um, most mornings and get up for breakfast now… I definitely am going to see friends more now … I'm definitely talking to other family more often.

Other AOI group participants described how making even small changes had a flow on effect, that is, how one action led to another, and then another, as self-belief and confidence grew. In John's words:

Small thing of going next door to have coffee, which was an action, increased self-esteem and confidence, then I kept going to groups … going to church every Sunday.

Shandy too described choosing a quick activity change within the group that led to other benefits: “I started going for coffee and stuff,” which made her feel, “ah, fantastic.” She then went on to describe the benefit of being with others, who were similarly stuck and experiencing similar challenges in getting going, and the changes that she had made from AOI group participation:

[AOI] group helped put some actions in place … I like to see more people, I like to do things with people and my last comment, I enjoy activities … I take care of myself a bit more.

Listening to other peoples' opinions actually brings the motivation out of my own … yep, it helped motivate me.

#### Recognizing the Value of Meaningful Activities

Following completion of the AOI groups, participants identified activities that were personally meaningful to them, and how they had developed ways of participating in them. For some AOI participants this involved reconnecting with, or remembering the meaning of previous activities; for others, it reflected gaining further understanding of how meaningful activities connect with health and well-being. For example, participating in the AOI group assisted James to “recognize [the] importance of activities which has made a change in life.” Further, Frank illustrated how, through the AOI group, he gained a different perspective of what was important to him within his daily routine:

It's just like this. You wake up in the morning. You go to the toilet, have a shower. Now, you know… you think self-care. It has to happen because it's a way of caring for yourself, you know. … Because, normal day-to-day living, [before] I didn't use much time to think about these things I'm doing every day, normal routine things. What are the definitions of them … and that's kind of soothing… After attending the group, I am now about to put some perspective into what is going on in my life.

In identifying activities that were meaningful to them, AOI participants also understood more about how those activities contribute positively to their lives, influencing their emotions, confidence, sense of well-being, and motivation:

I've been more confident … a bit more relaxed … fairly happy with the group. Probably [doing] more activities. [Craig]

… gives you confidence, very well, self-esteem, more cheerful … I feel happy. [James]

#### Doing Things Brings a Sense of Hope and Recovery

AOI group participants noted that being engaged in doing things made them feel like their lives were going in a positive direction. For example, James shared how participation in daily activities enabled him to “develop new skills and knowledge” and to “feel as though I have accomplished something,” so that being at the CCU and participating in AOI had been a step in making “recovery a tiny bit faster.” For Shandy too, her experience of the AOI group supported her orientation toward positives and the future:

We just mainly focused on our future … so looking toward the goals and what we would like to achieve and stuff … It's just getting out there, having a chance to do things with other people and doing things for myself, makes me feel good.

When asked directly about recovery, most AOI participants responded by offering a future perspective—describing how their lives would be when they “recovered.” This was illustrated by John, who had been “working on achieving goals since participation in group,” and was back at school completing an adult literacy, computer and spelling course as a result. James too described how he has learnt to think about recovery, drawing connections between what he does and how he feels, while Shandy valued talking about recovery and what being healthy meant to her during the AOI group, noting it helped to “motivate me”:

I think just putting it out there actually pushed me a little bit further to achieve my goals … I just like how we all got together and we were all able to speak our minds.

Further, in relation to her recovery, Shandy also described how using the daily activity chart informed her about both what she was doing and how her activities were impacting her: “it was good doing that.” Other participants too shared ways in which participating in AOI group supported their sense of moving forward. For example, John recalled one of the group activities to illustrate how he was changing his life:

… it has changed a lot … [occupational therapist] had three pictures. The first one was a man sitting on a couch. He did not have a shower, was a bum and was not doing anything and the second one was of a man in the office just stressed out and all that paperwork he has to do, and the third one was a man at the supermarket and he was all smiling and cleaned and all this. We had to figure out which one was us and all this. I picked man on couch not wanting to do anything … That used to be me, I used to do things like this … Now I'd pick the man in the middle on the shopping trolley. I have gone to group, go to school Mondays … it feels good.

In addition, while being in the AOI group, doing and talking about activities together supported participants' sense of recovering, so did participating in activities that created a sense of belonging and contributing in the social world:

Interacting socially and playing sports helps to make [me] feel better. [Craig]

Just getting out there and having a chance to do things with other people and for myself … Action wise, well I've started going for coffee and stuff. [Shandy]

And doing something useful for others “definitely makes me feel as though I am making a valuable contribution to society” [James].

### Facilitating Change

Recognizing inertia as a challenge, getting people going and looking at how AOI works to impact inertia were key themes that influenced the occupational therapists in facilitating change. Table 3 summarizes these themes.

**Table 3:** “Facilitating change”—a summary of key themes and descriptions.

**Key theme**	**Description**
Recognizing inertia as a challenge	1. Understanding of inertia. 2. Impact of inertia on participation. 3. Environment culture and expectation. 4. Staff being influenced by inertia.
Getting people going	1. Developing an understanding of what was assisting AOI participants to get going. 2. Impact of participation in the group raising AOI participants' awareness.
How AOI works to impact inertia	1. Impact of participation in group. 2. Challenges of converting AOI. 3. Fitting AOI content to participation. 4. Facilitation.

#### Recognizing Inertia as a Challenge

The AOI facilitators identified inertia as a challenge experienced by many AOI group members. They also described it as pervasive in its nature and holding sway over the overall culture within the CCUs, translating into environments of low expectation and limited activity participation. As Jess and Josh reflected, the “sense of inertia is very widespread,” as well as the degree of “stuckness” and lack of movement experienced by some residents. In Jess' words:

… they were really struggling to like get involved in different activities and stuff, or they would just kind of start something and stop and yeah, wouldn't be able to kind of make those long-term changes.

As a consequence, the AOI facilitators believed that AOI held potential value as a structured way to address these challenges within the CCU context. In Josh's words:

A big kind of reflection from me [is] that this type of group actually needs to happen especially in a CCU space because that's why people are here, like there are problems. So in terms of the relevance, there is a huge relevance. There is no way you can pretend that this is not relevant.

While the AOI facilitators recognized the disabling nature of inertia, they also found it was not immediately obvious to the AOI participants. They described having to work at exploring ideas around inertia and how this impacted health and well-being, and contrasting this with the positive impacts of activity (or Action Over Inertia). For instance, Sam found that revisiting the ideas regularly in the groups facilitated AOI group participants to understand more fully, and to make connections with their own lives and daily routines.

#### Challenges of Getting People Going

Getting people going in the CCU environment was a challenge experienced by all AOI facilitators. From their perspectives, this stemmed from a range of factors, including perceived low service expectations of residents' active participation and skill development, as well as the challenges of inertia or stuckness on the part of residents. Running AOI groups also challenged facilitators' own ideas about CCU residents having skill deficits assumed either to be cognitive or symptom-related in nature, and providing opportunities to reframe some of them as consequences of lack of opportunity to perform, practice and develop skills. For instance, having described initial reluctance to use worksheet-based activities during AOI groups, Josh noted: “A lot of clients don't do that day to day at all,” creating an unexpected opportunity for practice reading and completing forms that “turned out to be a real positive.”

#### Getting People Going

Similarly to AOI group participants, the facilitators spoke about the “stuckness” and difficulty in “getting people going” and acknowledged the challenges that AOI participants experienced, while also describing the groups as supportive and encouraging toward each member.

Small changes and achievements were noticed and celebrated, with an awareness of the degree of challenge involved in getting going for some, if not all group participants. For example, as Sarah highlighted, being part of the group was evidence of change for one AOI participant: “We had someone who would just do the very bare minimum, but for him to be in the group was the biggest achievement.” Josh too highlighted the importance of other CCU staff also noticing subtle changes in a participant's engagement in activities and interactions with others. Further, as Jess described, it was “rewarding to see some of participants, and the changes they made during the eight week period.”

Several AOI facilitators noted the value of AOI worksheets for sharing ideas relevant to the lives of group participants and communicating them in effective ways, as Josh described:

I was constantly thinking about how would I bring this up and I would talk about, you know, my own experience or try to give a little anecdote or a story or something, or think of a question to prompt people to give me an answer or something to kind of lead the discussion.

Sam further noted the benefits of the AOI group format, noting that participants gave “more detailed responses when able to share in a group, could bounce off ideas and relate to one another.” She also described how the group seemed to generate its own energy, having a positive flow on effect on the motivation of individuals: “in a group setting you could see motivation levels really increase, especially with quick activity changes.” Further, Snoopy reflected that, by the end of the AOI group, it “opened people's eyes to what they were doing with their time,” and the role of activity in recovering:

There seemed to be a shared understanding that being active and involved in meaningful activities—that it is an important part of recovery and is not just something that you do separate to having a mental illness.

#### How AOI Works to Impact Inertia

AOI facilitators also identified a range of factors that made it hard to convert talking about AOI ideas into real changes in AOI participants' lives and activity patterns. The AOI workbook was designed for flexibly tailored use, and AOI facilitators adjusted the ways in which they shared the content within groups to ensure its relevance for participants, including by turning worksheets into activities; using examples; providing one-on-one support; and asking questions that could lead to meaningful discussion. For example, Snoopy used:

Pictures and got participants to put where they were on the scale. [This] helped people to engage more, and pick things out of the box, and talk more. … So, I think it was helpful doing things on the whiteboard, getting people to write up things a bit more … just thinking about what kind of tactile kinds of things people can do to keep their attention.

The AOI intervention is about both understanding and acting on barriers to change, including use of coaching to support “*in-vivo*” activity change. So, while the group format facilitated knowledge and understanding of barriers to activity participation, the facilitators had the sense that this alone was insufficient to facilitate lasting change. Overall, the group setting was seen as facilitating the AOI intervention in that it provided an environment for common themes to emerge around barriers to change, peer learning, and opportunities for group members to bounce ideas off each other. Hence, in Snoopy's words, participants were “able to identify pros and cons of different occupational balance or imbalance, and they were able to review their daily time use and get a sense of what their just right balance would be.”

Peer learning and support were also valuable aspects of AOI groups from a facilitators' perspectives. For instance, Sam described how peers listening to and learning the benefits gained from being more active from each other was motivating, and led to offers of support: “one group member offered to assist someone else because that was a strength of theirs.” Sam also elaborated:

I think if done individually, the motivation, the willingness to do it wouldn't have been as high as compared to that group setting. … I think you got a lot better response or more detailed responses when they were able to share in a group'.

On the other hand, learning in a group setting could also potentially be confronting, as Snoopy reflected:

… one participant [was] doing a lot with his time and I wonder whether that was challenging for other clients to hear… comparing themselves to him and maybe feeling a bit lacking perhaps. But then at the same time, maybe that was a positive too because they got to see a peer of theirs that was quite active and that he was obviously getting a lot of benefits from being active.

Challenge if people [are] feeling defensive about lack of participation as it was more public for them. Did open peoples' eyes to what they were doing with their time, which was difficult for people to acknowledge.

Nevertheless, by the end of the AOI group intervention, the facilitators noted overall a “sense of client understanding … it led to people being more motivated about activity levels” [Snoopy]; having “changed their mindset” [Josh]; being “really proud of self” [Jess]; and sharing and celebrating “smaller steps toward the change” with others in their group [Sam]. In their overall reflections, the facilitators emphasized needing to understand each group participant, so they could tailor group implementation to optimize each person's engagement. This knowledge allowed them to offer tailored suggestions to CCU staff for enabling activity engagement, so as to break the sense of inertia in the CCU environment.

## Discussion

This qualitative case study research is the first known to report the use of Action Over Inertia (AOI) within community residential rehabilitation services for people with enduring mental illnesses in Australia. Action Over Inertia (AOI) is designed to support people with enduring mental illness in making change to disrupted or restricted activity patterns that limit their opportunities for health and well-being benefits (25). The findings of this study suggest AOI works because it allows a re-conceptualizing of inertia for both participants and facilitators or service providers. The issue of “inertia” is itself not new, issues with energy and drive have long been associated with schizophrenia (40). Furthermore, Deegan (41) described the despair experienced by people whose lives are characterized inertia and the important role of supporters to instill hope of a future beyond inertia. Others too have identified the ways in which not only cognitive intrusions and disruptions, but also failed efforts to make sense of experiences and overcome difficulties can spiral into a diminished sense of agency and capability, withdrawal and demoralization (42, 43). Hence, as the group participants in this study identified, inertia or stuckness is real and difficult to overcome, but neither an intractable symptom nor insurmountable with the right support. That participants identified making activity changes as hard and confronting, albeit rewarding, is consistent with Bjørkedal et al.'s (44) findings of a previous evaluation of occupational therapy to enable re-engagement in activities during recovery from participant perspectives. Yet, the findings of this study indicate AOI appears to support people to explore “inertia” and consider alternative ways of understanding this “stuckness” and the barriers to “getting going,” which in turn makes imagining change possible.

Imagining change as possible aligns with both instilling hope and the recovery process (4, 9, 42). Findings from this study indicate that both AOI participants and facilitators gained greater awareness and understanding of this sense of “stuckness,” enabling the development of hope and a sense of agency, or feeling of “I can do something about this” on both their parts. As a consequence, AOI participants started taking small steps in making self-directed activity changes that increased their self-efficacy and confidence. This improved sense of capability in turn led to doing more, and feeling more capable and less “stuck.” A similar process of gaining momentum has also been described elsewhere (31, 42, 45). In turn, this process created possibilities for staff to reframe previous low expectations as a lack of opportunity to perform, practice and develop skills, so that barriers to activity participation might be viewed as more complex than previously understood, and not solely about negative symptoms or cognitive difficulties. As highlighted by McKenna et al. (2) and Muerk et al. (46), CCUs are in a transition from a clinically-oriented rehabilitative model of care to one informed by a recovery framework. In this context, the competing perspectives of clinical and personal recovery perspectives may contribute to underlying low expectations or pessimism about CCUs residents' potential for recovery. The latter study by Muerk et al. explored an integrated staffing model involving peer support and clinical staff, highlighting the potential of bringing together lived experience and therapeutic perspectives to foster recovery-promoting environments within Australian CCU service settings. Given the present study indicates Action Over Inertia groups can facilitate greater understanding of inertia and activity engagement as experienced by consumers, this suggests further collaborative development and co-facilitation of AOI groups with peer support workers offers promise for strengthening their contribution to recovery focused practice in CCU settings.

This present study is also novel in describing the use and value of AOI as a group intervention. The findings indicate that learning occurred for both participants and facilitators as they engaged with the content and structure of the AOI groups. Action Over Inertia is a workbook-based resource intended for use in collaboration with people experiencing with severe mental illness, so as to offer individualized support for engagement in activity patterns that promote recovery and well-being (25). The factors that disrupt activity patterns and restrict engagement in meaningful activities are multiple and intersecting. Thus, Action Over Inertia is neither prescriptive, nor primarily education focused, recognizing that enhanced knowledge about connections between activity patterns and well-being and about barriers to change, while useful, is not sufficient to enable lasting change in activity patterns, as participants in this study also indicated. The “doing” within the group was critical in cementing ideas and supporting participants to try them out and see that change was possible. This speaks to the interactive process between action and belief (31).

The social dimension of this approach should also not be discounted: the group provided participants with a supportive structure for building awareness, for experimenting with making changes and reinforcing success; a counter experience to tackling and failing at large goals alone (31). As in other activity change focused groups (29, 47), the AOI facilitators used participation in activities along with social interactions between members to educate, inspire, and instill hope. Similarly, Lund et al. (29) described the power of the group for supporting change, highlighting that group participants gain value from connecting with and helping each other, the sense of belonging and the provision of mutual support in groups, as well as the content of the group. Hence, all AOI facilitators used the AOI manual (25) and locally-developed AOI group intervention guide, but they also recognized the need to not only familiarize themselves with the content but also to apply it with creativity and flexibility in structuring the group sessions to address the concerns and interests of participants over the course of group sessions. The need to tailor manualized and structured psychosocial interventions to participants' particular needs, aspirations and contexts is increasingly recognized as necessary to bring about health-enhancing changes in people's everyday lives, along with ensuring ongoing support (47). Offered in a group format with community residential rehabilitation settings, such as CCUs, Action Over Inertia can provide group support for self-development and self-directed change in patterns of everyday activities that promote recovery. Furthermore, collaboration with peer support workers could enhance the ongoing support available to sustain changes beyond the groups themselves so as to support experiences of inclusion and citizenship, presently the least well-developed among recovery oriented practices (4).

### Study Limitations

While this is a small-scale study, the inclusion of data from multiple sources and across three service sites and two time-points reflects adherence to case study research methodology and enhances the authenticity of its findings (33). Typically, qualitative case studies will have limited applicability beyond their specific setting studied, so that conclusions drawn about their wider generalisability need necessarily to be modest. A detailed description has been provided of the Action Over Inertia intervention and the Community Care Unit setting in which it was implemented, so as to allow readers to judge the applicability of the results to another setting for themselves (48). In regards to the intervention itself, while reported experiences were generally positive, a 6- to 8-week group program may be unlikely to enable sustained change for people with long-standing patterns of disengagement from activities or disrupted activity patterns. Hence, studies that further investigate AOI related change processes and outcomes over longer timeframes should be a priority, with changes in time use or activity patterns, meaningful engagement in occupations, and recovery related outcomes that matter from consumer perspectives given particular attention.

## Conclusions

Involvement in self-chosen, personally and culturally meaningful activities is a recognized contributing factor in recovery and well-being (11, 27), so that the further development of effective approaches to individualized support for engagement in meaningful and healthy activity patterns is important (21). This is the first known attempt to explore the use of one such approach, Action Over Inertia (AOI), in Australian community residential mental health rehabilitation services. Further, it is novel in its description of AOI as a group-based intervention. The findings indicated that AOI provided Community Care Unit residents with valued support to identify barriers to more active living, to appreciate the connections between their time-use, health and well-being, to reframe inertia and take steps to overcome it. The group facilitators too gained a stronger appreciation of the importance of recognizing inertia as a challenge in facilitating people to effect change in their lives. Action Over Inertia offers a flexible approach that provides tools and resources to promote meaningful and healthy patterns of activity engagement as part of recovery oriented practice, the benefits of which merit further research in collaboration with adults receiving community mental health services.

## Data Availability Statement

The datasets presented in this article are not readily available because ethical approval for wider sharing of the original datasets was not granted in the interests of protecting participants' privacy and confidentiality. Requests to access the datasets should be directed to Ellie Fossey (ellie.fossey@monash.edu).

## Ethics Statement

This study involving human participants was reviewed and approved by The Institutional Human Research Ethics Committees (HRECs) of Melbourne Health (2015.103) and La Trobe University Faculty of Health Sciences (LEG/13218) approved this research. The participants provided their written informed consent to participate in this study. Written informed consent was obtained from the individual(s) for the publication of any potentially identifiable images or data included in this article.

## Author Contributions

ER undertook this research as part of her Masters of Advanced Occupational Therapy, designed the study, with support and guidance from the co-authors, interviewed the participants and analyzed the data, and drafted the initial manuscript. PE and EF supervised the research process, contributed to the manuscript development, reviewed and added material to manuscript drafts. All authors contributed to the article and approved the submitted version.

## Conflict of Interest

The authors declare that the research was conducted in the absence of any commercial or financial relationships that could be construed as a potential conflict of interest.
